# Mental Health of University Students During and After the War: Battle for Ukrainian Future

**DOI:** 10.1192/j.eurpsy.2025.142

**Published:** 2025-08-26

**Authors:** V. Seleznova

**Affiliations:** Economics, Taras Shevchenko National University of Kyiv, Kyiv, Ukraine

## Abstract

**Objective:**

The study aims to analyze the mental health conditions of Ukrainian university students during and after the war and to examine the impact of these conditions on their academic productivity.

**Methods:**

The research combines methods of cross-sectional surveys and regression analysis. The survey includes a demographic section, a mental health screening, and the **“**Work Productivity and Activity Impairment: Specific Health Problem**”** (WPAI: SHP) section, adapted for the study’s purposes. The sample consists of 1,398 respondents.

**Results:**

Among all respondents, 85.8% exhibited symptoms of depression, 66.1% of anxiety disorder, 56.9% of sleep disorders, and 48.1% of post-traumatic stress disorder (PTSD). Econometric modeling results confirm the devastating impact of mental health issues on students’ academic productivity during the war: moderate to severe symptoms of mental disorders, such as depression, anxiety disorder, and sleep disorders, are associated with a decline in students’ productivity by 17.4%, 12.2%, and 11.0%, respectively.

**Conclusion:**

The prevalence of mental health issues among university students during and after the war highlights an urgent need for interventions to support their well-being and academic performance. These efforts are critical for fostering a resilient and capable generation essential for Ukraine’s postwar recovery and future development.

**Keywords:**

Mental health, mental conditions, war, academic productivity, students.

**Disclosure of Interest:**

None Declared

